# Association of radiation risk in the second and third generations with polymorphisms in the genes *CYP1A1, CYP2E1, GSTP1* and changes in the thyroid

**DOI:** 10.1186/s10020-019-0117-y

**Published:** 2019-11-14

**Authors:** Meruyert Massabayeva, Nailya Chaizhunusova, Nurlan Aukenov, Tolkyn Bulegenov, Bakytbek Apsalikov, Aigerim Shapihanova, Yersin Zhunussov

**Affiliations:** 1 Medical University Semey, Semey, Kazakhstan; 2 Department of Health and Human Resources, Ministry of Health of the Republic of Kazakhstan, Nur-Sultan, Kazakhstan

**Keywords:** Radiation risk, Second-third generations, *CYP1А1*, *CYP2Е1*, *GSTP1* genes polymorphisms

## Abstract

**Background:**

To study the association of radiation risk in the 2nd –3rd generations with polymorphisms in the genes *CYP1A1, CYP2E1, GSTP1* and changes in the thyroid.

**Methods:**

5 polymorphic gene variants (rs1048943, rs4646421, rs2070676, rs3813867, rs1695) were studied in 399 people living in the East Kazakhstan region in this research. 248 people of the 2nd - 3rd generation lived in the territory with radiation exposure in Abai, Borodulikha areas, and 151 people the comparison group lived in Kurchum district without radiation exposure comparable in sex and age with control group.

**Results:**

The results show that there is a significant association of rs1048943 in exposed and unexposed groups (*p* < 0.003), and the absence of association of rs4646421, rs2070676, rs3813867, rs1695 in the studied groups. The mean value of thyroxine in carriers of the AG + GG genotype of rs4646421 is significantly lower than in AA genotype carriers (*p* = 0.04); no significant changes were found in genotypes’ distribution with thyroid-stimulating hormone and anti-thyroid peroxidase indicators. Significant changes were in levels of anti-thyroid peroxidase between exposed and unexposed groups (*p* = 0.007). The thyroxine - thyroid-stimulating hormone levels were not significantly different in exposed and unexposed groups (*p* > 0.3).

**Conclusions:**

This study demonstrated the association of rs1048943 polymorphism with living in the radiation zone in the 2nd and 3rd generations for the first time. Thyroxine levels decrease was identified in the 2nd and 3rd generation residents of the exposed area, as well as a significant increase of anti-thyroid peroxidase occurs in individuals of the 2nd and 3rd generation living in areas with radiation exposure**.**

## Introduction

The impact of environmental factors affecting the health of the population is an urgent problem at the present time. An intense anthropogenic impact on the environment leads to the emergence of environmentally caused human diseases, which can lead to dysfunctions of various body systems. One of the unfavorable factors include ionizing radiation (Markabayeva et al., 2018). Many works have been published recently on the medical consequences of long-term radiation effect on the health of directly exposed people and their descendants which allowed to establish a significant increase in somatic pathology, changes in the immune status, genetic effects of radiation-induced factors (Tanaka et al., 2000; Mudie et al., 2007; Kolyado et al., 2019). Hence, (Tang and Loganovsky, 2018) stated that exposure to low doses of ionizing radiation can affect health, depending on the genetic background, gender, age and source of radiation.

East Kazakhstan region (Kazakhstan) applies to the territories with radiation burden (Grosche et al., 2015). The population living in this area had been exposed to various doses of radiation for a long time (Simon & Bouville, 2002). The risk assessment of the effect of radiation exposure on individual organs or the body as a whole is based on an in-depth study of mechanisms of molecular and biochemical changes in the body. It has been established that exposure to radiation depending on the dose and age increases the risk of developing thyroid cancer (Yamashita et al., 2017). Survivors of the atomic bombing in Hiroshima and Nagasaki had a higher risk of radiation-induced thyroid cancer in children and adolescents (Furukawa et al., 2013). Genetic predisposition to thyroid diseases should be distinguished from hereditary genetic pathologies because its basis is the genetic polymorphism inherent in humans, but not the mutations of certain genes (Saenko & Rogounovitch, 2018).

Genes encoding enzymes 1st and 2nd phase of the process of biotransformation of foreign chemicals, carcinogens, environmental pollutants and other xenobiotics include *CYP1A1, CYP2E1, GSTP1* (Nourozi et al., 2018). Mutations in the structure of these genes lead to an increase in the concentration of intermediate toxic metabolites in the body and the accumulation of free radicals (Caccamo et al., 2013).

This study was conducted to investigate the association of radiation risk with polymorphisms in the genes SUR1A1, SUR2E1, GSTP1 and changes in the thyroid gland in individuals of 2nd - 3rd generations exposed to low doses of radiation and parents/ grandparents who lived during the nuclear tests.

## Methods

### Subjects

A total of 399 people living in the East Kazakhstan region (EKR) took part in the study. The contingent of surveyed persons included 248 people living in areas with adverse environmental factors, such as radiation exposure (Karaul village, Abay district and Borodulikha village, Borodulikha district, East Kazakhstan), as well as 151 people were not exposed to adverse environmental factors (Kurchum village, Kurchum district, EKR). These areas were included in the study due to different radiation doses as a consequence of the localization of areas at different distances from the Semipalatinsk Test Site (STS). The State Scientific Automated Medical Register (SSAMR) serves as a mechanism for assessing the individual dose of radiation received by a person while living in the territories adjacent to the STS between 1949 and 1990. Persons aged from 27 to 50 years living in the territory of Abay district of East Kazakhstan received individual dose loads in the range from 0.06 to 17 cSv due to the activities of the STS. For the population of Borodulikha district of East Kazakhstan, this range was from 0.05 to 15 cSv. Kurchum district of East Kazakhstan is environmentally safe. According to Article 6 of the 2nd chapter of the Law of the Republic of Kazakhstan of December 18, 1992 N 1787-XII “On social protection of citizens who suffered from nuclear tests at the Semipalatinsk nuclear test site”, Abay district belongs to the zone of maximum radiation risk, and Borodulikha district belongs to the zone of increased radiation risk. In turn, Kurchum district which is not mentioned in this law is chosen as a zone for the comparison group. The population of the above-mentioned territories were taken from the database of the State Scientific Automated Medical Register (SSAMR) of the population of Kazakhstan exposed to radiation (Grosche et al., 2015).

Criteria for inclusion: age 18–60 years (2 – 3rd generation with exposure), legally confirmed residence of parents (grandparents) in the territory of the influence of Semipalatinsk Test Site during nuclear weapon tests (in accordance with the database of the SSAMR of the population of Kazakhstan exposed to radiation); and persons permanently residing in the territory of the Kurchum district, EKR. Exclusion criteria: organic CNS damage, somatic diseases in the stage of decompensation, the presence of genetic diseases.

### Genotyping and thyroid hormone levels analyses

Laboratory measurements of thyroid hormones were carried out in the laboratory In Vivo, Semey, Kazakhstan by Chemiluminescent enzyme immunoassay (CLIA) method on the equipment Architect i2000SR (Abbott Laboratories, IL, USA) using commercial diagnostic kits (Abbott Laboratories, USA) in accordance with the manufacturer’s instructions. Indicators of the reference ranges for thyroxine are (T4) – 9-22 pmol/L; thyroid stimulating hormone (TSH) – 0.4 – 4.0 mIU/L; antithyroid peroxidase (anti-TPO)-0–35 IU/mL.

For genetic research, peripheral blood taken into vacuum tubes with K2/K3 EDTA of subjects was used. Isolation of genomic DNA from blood samples (100 μl) was performed using ready-made commercial kits GeneJET Mini kit (Thermo Scientific, Vilnius, Lietuva), carefully in accordance with the manufacturer’s instructions. DNA concentration was assessed using fluorometer Qubit 4 (Thermo Scientific, Waltham, MA, USA). The isolated DNA was frozen and stored at − 20 °C.

Genotyping of 399 samples was carried out by PCR in real time on the CFX 96 (BioRad, CA, USA) device using ready-made mixtures of primers and TaqMan probes (catalog numbers: 4362691 for rs1048943, rs1695, rs3813867 rs2070676; and 4,351,379 for rs4646421) in the presence of TaqMan Genotyping Master mix reagent and 40 ng DNA as a matrix in the total volume of 20 μl in 96-well plates (all reagents produced by Life Technologies, Foster city, CA, USA). The amplification program included pre-denaturation at 95 °C for 3 min, followed by 48 cycles at 95 °C for 10 s and 60 °C for 40 s for all SNPs.

### Statistical analyses

Statistical analysis was performed using IBM SPSS Statistics Version 21 (International Business Machines Corp., Armonk, NY, USA).

Comparison of genotypic distribution frequencies between groups was performed using χ2 criterion (Pearson’s chi-squared test). Continuous variables were assessed for normality using Shapiro–Wilk test, as well as the Mann-Whitney or Kruskal - Wallis quantitative test was utilized for one-dimensional analysis. Logistic regression tests were used to determine the independent influence of radiological history; thyroid parameters were used as dependent variable. However, the influence of gender and age was not taken into account in the logistic regression. Differences between samples were considered statistically significant at *p* < 0.05. Deviations of genotype frequencies of each polymorphism from the Hardy-Weinberg equilibrium were estimated by χ^*2*^ − test.

## Results

The demographic characteristics of the subjects are presented in Table 1. A total of 399 people of which 248 people with radiological history (2 – 3rd generations with exposure) and 151 persons the control group without radiological history. Among exposed people only 19.5% were male and 80.5% female, similar data was among unexposed people: 21% were male and 79% were female. The studied groups are comparable by sex and age.

**Table 1:** Demographic Characteristics According to Exposed and Unexposed Groups (*N* = 399)

	Exposed (*n* = 248)		Unexposed (*n* = 151)	*P* value
	Exp1 (*n* = 118)	Exp2 (*n* = 130)		
Males, *n* (%)	42 (28)	15 (11)	32 (21)	0.974
Age (years)	18–60 36.28 ± 7.3	18–58 38.0 ± 7.0	18–58 38.0 ± 7.0	1.0

Exp1: Abay District, East Kazakhstan

Exp2: Borodulikha District, East Kazakhstan

Unexp: Kurchum District, East Kazakhstan

Table 2 shows the genotype frequency of polymorphisms in the genes CYP1A1 (rs1048943, rs4646421), CYP2E1 (rs2070676, rs3813867), GSTP1 (rs1695) in the exposed (Abay and Borodulikha districts) and unexposed (Kurchum district) groups. Significant difference in the distribution of rs1048943 polymorphism genotype in the exposed and unexposed groups (*p* < 0.003) (Table 2). It should be noted that significant differences were both in the comparison of the exposed Abai district with the unexposed Kurchum district, and in the comparison of Borodulikha district with the latter. There were no statistically significant differences in the distribution of genotypes of polymorphisms rs4646421, rs2070676, rs3813867 and rs1695 (*p* > 0.004). No deviations from the Hardy-Weinberg equilibrium were found in any group (*p* > 0.05).

**Table 2:** Genotype Frequencies of Polymorphism CYP1А1, CYP2E1, GSTP1 genes in the studied groups

SNPs	Exp1 (%)	*P* value	Exp2 (%)	*P* value	VS Unexp (%)
1*.CYP1А1* rs1048943					
AA	67 (59)		68 (70)		44 (33)
AG	36 (32)	0.0002*	24 (25)	0.0002*	73 (55)
GG	11 (9)		5 (5)		15 (12)
2. rs4646421					
AA	29 (25)		27 (23)		33 (25)
AG	47 (41)	0.97	47 (41)	0.92	55 (41)
GG	39 (34)		42 (36)		45 (34)
3. *CYP2Е1* rs2070676					
CC	85 (76)		86 (69)		99 (79)
CG	22 (20)	0.89	33 (26)	0.17	22 (18)
GG	4 (4)		6 (5)		4 (3)
4. rs3813867					
CC	17 (15)		21 (17)		8 (5)
CG	45 (39)	0.02	47 (38)	0.004	55 (37)
GG	53 (46)		55 (45)		86 (58)
5. *GSTP1* rs1695					
AA	84 (74)		82 (77)		79 (64)
AG	24 (21)	0.23	20 (19)	0.09	35 (29)
GG	5 (5)		4 (4)		9 (7)

* *p* < 0.003 with correction for the multiplicity of comparisons

*SNP* single nucleotide polymorphism

Table 3 demonstrates the association between the genotypes of *CYP1A1* (rs1048943, rs46421), *CYP2E1* (rs2070676, rs3813867), *GSTP1* (rs1695) and thyroid parameters (thyroxine, thyroid-stimulating hormone, anti-thyroid peroxidase) in the studied populations. Since few of them had the genotype AG and GG in the polymorphisms rs1048943, rs46421, rs1695 we combined them AG + GG, we also combined the genotypes CG + GG of polymorphisms rs2070676 and rs3813867. The average value of T4 in carriers of АА (11.7), AG + GG (8.9) polymorphism rs4646421 was significantly lower in residents of Abay district than in residents of the control Kurchum district АА (12.0), AA+AG (11.8) (*p* = 0.04); however, there were no significant changes in the distribution of genotypes with TSH and anti-TPO levels. We did not find a statistically significant association between the polymorphisms rs1048943, rs2070676, rs3813867, rs1695 and parameters of the thyroid gland (*p* > 0.05) for any comparison groups.

**Table 3:** The Association between Genotypes and Thyroid Hormone Parameters in the Studied Population

	SNPs	T4 Me (Q1- Q3)	TSH Me (Q1- Q3)	anti-TPO Me (Q1- Q3)
Exp1	rs1048943 AA AG + GG	11.6 (10.7–12.5)	2.0 (1.3–3.0)	0.4 (0.1–4.3)
	rs4646421 AA AG + GG	11.7 (10.8–12.5)*	2.0 (1.3–3.0)	0.4 (0.1–3.5)
	rs2070676 CC CG + GG	11.7 (10.8–12.5)	2.0 (1.3–3.0)	0.4 (0.1–6.8)
	rs3813867 CC CG + GG	11.7 (10.8–12.5)	1.9 (1.3–2.9)	0.4 (0.1–3.5)
	rs1695 AA AG + GG	11.7 (10.8–12.5)	2.0 (1.3–3.0)	0.4 (0.1–5.1)
Exp2	rs1048943 AA AG + GG	12.2 (11.5–13.1)	1.8 (1.3–2.8)	0.3 (0.2–0.9)
	rs4646421 AA AG + GG	12.3 (11.5–13.2)	1.6 (1.1–2.8)	0.3 (0.2–0.9)
	rs2070676 CC CG + GG	12.3 (11.5–13.2)	1.7 (1.1–2.8)	0.4 (0.2–0.9)
	rs3813867 CC CG + GG	12.2 (11.5–13.1)	1.6 (1.1–2.8)	0.3 (0.2–0.8)
	rs1695 AA AG + GG	12.3 (11.6–13.2)	1.8 (1.3–2.9)	0.3 (0.2–0.8)
Unexp	rs1048943 AA AG + GG	12.0 (11.3–13.2)	1.7 (1.2–2.9)	0.3 (0.2–0.9)
	rs4646421 AA AG + GG	12.0 (11.1–13.0)	1.7 (1.2–2.9)	0.3 (0.2–1.3)
	rs2070676 CC CG + GG	12.2 (11.3–13.3)	2.0 (1.0–3.0)	0.4 (0.2–0.9)
	rs3813867 CC CG + GG	12.0 (11.3–13.1)	1.7 (1.2–2.9)	0.3 (0.2–1.3)
	rs1695 AA AG + GG	12.2 (11.3–13.3)	1.3 (1.0–2.0)	0.3 (0.2–0.3)

* *p* < 0.05

Table 4 presents logistic regression data to analyze the association between radiological history and thyroid parameters. Significant changes were identified in anti-thyroid peroxidase levels between exposed and unexposed groups (*p* = 0.007). Indicators of thyroxine, thyroid-stimulating hormone were not significantly different in exposed and unexposed groups (*p* > 0.3).

**Table 4:** Associative Analysis of the Radiological History and Parameters of the Thyroid Using the Linear Regression Model

Dependent variable	Radiological History		
	Вeta	Se	*P* value
T4	0.000	0.000	0.314
TSH	0.007	0.057	0.909
anti-TPO	0.440	0.164	0.007*

*Note.* All correlations significant at *p < 0.05

## Discussion

Hereditary genetic traits are known to determine a person’s sensitivity to toxic and chemical substances (Ricarda Thier et al., 2003). The polymorphisms of genes encoding enzymes involved in the biotransformation of toxins and chemicals include cytochrome P-450 (CYP), glutathione S - transferase (GST) (Satyender Singh et al., 2011). In the present study, we found an association between rs1048943 polymorphism of *CYP1A1* gene with living in areas with radiation exposure in 2nd and 3rd generations, and there was no association of polymorphisms rs4646421, rs2070676, rs3813867 and *GSTP1* rs1695 with living in areas with radiation exposure.

In this study, we found an association between a polymorphism of the gene *CYP1A1* rs46421 and hormonal imbalance of the thyroid gland, a decrease in the level of T4 in carriers of the genotype АА in residents with radiation exposure of 2nd and 3rd generations of the Abai district of EKR, which belongs to the territory of maximum radiation exposure (Grosche et al., 2015). However, there were no significant changes in TSH and anti-TPO in Abai district. It is noteworthy that the average value of T4 and TSH coincides with the reference ranges (T4–9-22 pmol/L, TSH – 0.4-4.0 mIU/L) in the groups with and without radiation exposure, but the average value of anti- TPO in the group with radiation exposure is much higher than the reference ranges (anti-TPO – 0-35 IU/mL). The thyroid gland is known to be a radiation-sensitive organ (Suzuki S. et al., 2019). The risk of developing thyroid cancer also depends on the dose and has not been reduced for many decades (Williams et al., 2004; Tronko et al., 2017). It is worth noting that the EKR does not belong to iodine-deficient regions (Hamada et al. 2003), which in turn reduces the risk of thyroid disease, in contrast to the radiation exposure that is present in this study and can play a crucial role in the imbalance of thyroid hormones. Previous studies indicate that searching for new SNPs that may contribute to a genetic predisposition to thyroid disease is feasible synergistically with environmental studies (Saenko and Rogounovitch, 2018).

This is the first cross-sectional study of the search for a potential association of radiation exposure in individuals of the 2nd and 3rd generation with polymorphisms in the genes *СYР1А1* (rs1048943, rs4646421), *CYP2E1* (rs2070676, rs3813867), *GSTP1* (rs2070676, rs3813867) and changes in the thyroid gland in residents of EKR.

### Limitations

The study has some limitations. We recognize that we rely only on the value of T4, TTG, and anti-TPO, not including a more advanced thyroid study. In the future, we are planning a more detailed study of this issue with a large number of samples and a more thorough study of the thyroid gland. We also plan to continue this study with other genetic polymorphisms of enzymes involved in the metabolism of biotransformation of toxins and chemicals.

## Conclusions

For the first time the study revealed the association of rs1048943 polymorphism with living in the radiation zone in the 2nd and 3rd generations. Thyroxine levels decrease was found in the 2nd and 3rd generation residents of the exposed area, as well as a significant increase of anti-thyroid peroxidase occurs in individuals of the 2nd and 3rd generation living in areas with radiation exposure**.**

## Data Availability

The datasets used and/or analyzed during the current study are available from the corresponding author on reasonable request.

## Abbreviations

anti-TPO: anti-thyroid peroxidase
TSH: Thyroid-stimulating hormone
Т4: Thyroxine
